# Astaxanthin Alleviates Inflammatory Mechanical Hyperalgesia by Reducing Hyperexcitability of Trigeminal Nociceptive Secondary Neurons: Potential as an NSAID Alternative

**DOI:** 10.3390/molecules30183664

**Published:** 2025-09-09

**Authors:** Risako Chida, Mamoru Takeda

**Affiliations:** Laboratory of Food and Physiological Sciences, Department of Life and Food Sciences, School of Life and Environmental Sciences, Azabu University, 1-17-71 Fuchinobe, Chuo-ku, Sagamihara 252-5201, Kanagawa, Japan; f22001@azabu-u.ac.jp

**Keywords:** inflammation, trigeminal nociceptive neuron, hyperalgesia, pathological pain, extracellular single-unit recording, celecoxib, non-steroidal anti-inflammatory drugs, astaxanthin, carotenoid, complementary alternative medicine

## Abstract

This study investigated the potential of astaxanthin (AST), a natural carotenoid, to mitigate inflammation-induced hyperexcitability in the spinal trigeminal nucleus caudalis (SpVc) and the associated hyperalgesia. The efficacy of systemic AST application was compared to that of celecoxib (CEL). Inflammation was induced by injecting Complete Freund’s adjuvant into the whisker pads of rats. The mechanical escape threshold was then assessed by delivering mechanical stimuli to the orofacial region. Although inflamed rats exhibited a significantly lower mechanical threshold compared to naïve rats, this threshold was restored to normal levels two days after treatment with AST, CEL, and the 1/2 CEL + 1/2 AST combination. The activity of SpVc wide-dynamic range (WDR) neurons was measured using extracellular single-unit recordings in response to mechanical stimulation of the orofacial area under anesthesia. In inflamed rats, AST, CEL, and 1/2 CEL + 1/2 AST administration significantly reduced the average firing rate of these neurons elicited by both non-noxious and noxious mechanical stimuli. In addition, all three treatments significantly decreased the heightened average spontaneous activity of SpVc neurons and normalized the increased average receptive field size in inflamed rats. This study provides evidence that systemic AST administration attenuates inflammatory mechanical hyperalgesia. This action is associated with the suppression of hyperexcitability in nociceptive SpVc WDR neurons, likely through the inhibition of the cyclooxygenase-2 signaling pathway. These findings support the potential of AST as a therapeutic agent for complementary and alternative medicine. It may provide a valuable alternative to non-steroidal anti-inflammatory drugs for the prevention of trigeminal inflammatory mechanical hyperalgesia.

## 1. Introduction

Painful sensory signals from the orofacial region are transmitted by small Aδ-fibers and unmyelinated C-fibers of trigeminal ganglion (TG) neurons to second-order neurons located in the spinal trigeminal nucleus caudalis (SpVc) [1,2,3,4,5]. SpVc nociceptive neurons are commonly classified as either nociceptive-specific (NS) or wide-dynamic range (WDR) based on their responses to mechanical stimulation of the orofacial region, such as the facial skin. SpVc WDR neurons are notably distinguished by their responses to both noxious and innocuous stimuli [3,4]. The application of graded noxious stimuli to their receptive fields elicits a stimulus intensity-dependent increase in their firing frequency. The ability of WDR neurons to respond in a stimulus-intensity-dependent manner suggests their crucial role in encoding stimulus intensity. To model the trigeminal neural pathways that underlie pathological pain, researchers have developed rat models of orofacial inflammation using Complete Freund’s adjuvant (CFA). These models exhibit hyperexcitability of SpVc WDR neurons in response to mechanical stimuli [5,6]. Furthermore, SpVc neurons are implicated in the development of hyperalgesia and/or referred pain linked to dental pain [1,2,3,4,5,6,7].

The use of complementary and alternative medicine (CAM) has become increasingly prevalent in Western countries, particularly for patients with chronic pain who are refractory to conventional pharmacotherapy [8]. This trend suggests that CAM may have a role in the prevention of trigeminal inflammatory hyperalgesia. It is well-established that long-term consumption of dietary components, including polyphenols and carotenoids, can reduce inflammation-associated pain sensitivity. This action dampens the hyperactivity of SpVc WDR neurons by inhibiting both peripheral and central cyclooxygenase-2 (COX-2) cascade signaling pathways [9,10,11]. Taken together, these findings highlight the analgesic contributions of natural products derived from fish, vegetables, and fruits, especially for inflammatory pain.

Astaxanthin (AST), a naturally occurring carotenoid, is ubiquitous in living organisms, including plants, microalgae, crustacean shells (crabs, shrimps), and salmon [12]. In addition to being a more potent antioxidant than other carotenoids such as lutein and zeaxanthin [13,14,15], AST exhibits a wide range of biological activities [16,17,18,19]. Regarding excitable tissues, particularly the nervous system, a prior in vitro study has demonstrated that AST dose-dependently reduces glutamate release from rat cortical synaptosomes. This effect was attributed to the inhibition of presynaptic voltage-gated Ca^2+^ channels (Cav) and the mitogen-activated protein kinase (MAPK) signaling cascade [20]. Furthermore, Sharma et al. [21] reported that AST administration ameliorates neuropathic pain via its antagonism of N-methyl-D-aspartate (NMDA) glutamate receptors. Furthermore, AST is confirmed to cross the blood–brain barrier [22]. In our recent study, we revealed that acute intravenous administration of AST transiently inhibits trigeminal sensory transmission, including nociception, in models devoid of inflammatory or neuropathic pain. We propose that this effect is achieved through the inhibition of Cav channels and excitatory glutamate neuronal transmission, thereby positioning AST as a potential therapeutic agent for trigeminal nociceptive pain with a favorable adverse effect profile [23].

A study by Ohgami et al. [24] revealed that AST administration suppressed the concentration-dependent production of inflammatory mediators, specifically prostaglandin E2 (PGE_2_) and TNFα, in lipopolysaccharide-induced inflammation models (both in vivo and in vitro). Furthermore, AST inhibited the production of the PGE_2_-synthesizing enzyme, cyclooxygenase-2 (COX-2), in chondrocytes and microglial cells [25,26]. PGE2 induces pain hypersensitivity primarily by sensitizing various ion channels, including transient receptor potential ankyrin 1 (TRPA1), acid-sensing ion channels (ASIC), and voltage-dependent Na^+^ and K^+^ channels. This sensitization occurs through two distinct pathways: activation of protein kinase A (PKA) via prostanoid E (EP) receptors at nociceptive terminals [27], and a reduction in the activity of glycinergic inhibitory interneurons in the spinal dorsal horn [28]. A more recent study by Yajima et al. [11] demonstrated that chronic naringenin administration, involving a phytochemical found in citrus fruits, attenuates inflammatory hyperalgesia. This attenuation is achieved by reducing the hypersensitivity of nociceptive SpVc WDR neurons, which are responsible for encoding pain intensity, through the inhibition of the COX-2 signaling cascade predominantly at the inflammatory site. Moreover, naringenin has been confirmed to exhibit analgesic effects comparable to those of non-specific COX-2 inhibitors, exemplified by the non-steroidal anti-inflammatory drug (NSAID), diclofenac [11]. Diclofenac, a widely prescribed NSAID, possesses established analgesic, anti-inflammatory, and antipyretic properties, proving effective in managing a range of acute and chronic pain and inflammatory conditions [29]. Currently, to our knowledge, no studies have reported a relationship between food-derived chemical components and the specific COX-2 inhibitor, celecoxib (CEL) [30].

Collectively, these findings strongly suggest that AST administration mitigates inflammation-induced hyperexcitability of SpVc WDR neurons linked to trigeminal hyperalgesia, positioning AST as a potential therapeutic agent for preventing this condition. Importantly, to our knowledge, no prior studies have investigated whether systemic AST modulates nociceptive neuron excitability under inflammatory conditions. Consequently, employing behavioral and electrophysiological techniques, the present study sought to determine if in vivo AST administration attenuates inflammation-induced hyperexcitability of SpVc neurons, a condition linked to hyperalgesia in rats. Concurrently, we compared the suppressive potency of AST with that of the NSAID, CEL, a specific COX-2 inhibitor, on this hyperalgesia-associated, inflammation-induced SpVc neuronal excitability. Furthermore, we investigated whether substituting a half-dose of the conventional NSAID, CEL with a half-dose of AST could enhance the efficacy of CAM therapies while mitigating adverse effects. In this study, we found that AST administration attenuates inflammatory hyperalgesia associated with hyperexcitability of SpVc WDR neurons. These findings support AST’s potential as a therapeutic agent in CAM strategies for preventing trigeminal inflammatory mechanical hyperalgesia.

## 2. Results

### 2.1. The Development of Inflammation-Induced Hyperalgesia

**Figure 1:**
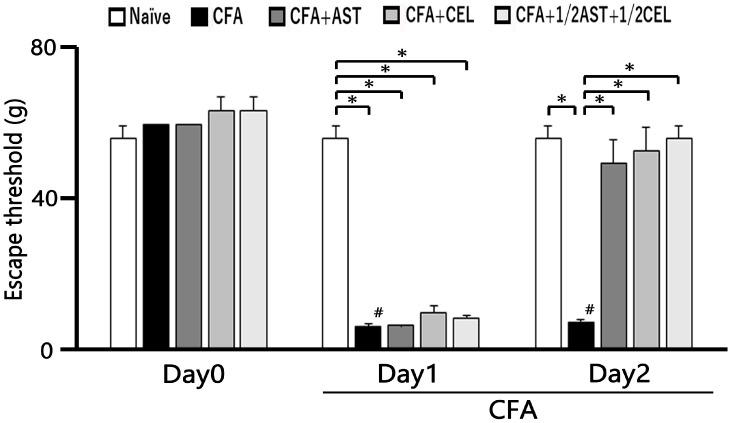
The escape threshold was measured and compared across five experimental groups: naïve, inflamed, inflamed with astaxanthin (AST), inflamed with celecoxib (CEL), and inflamed with 1/2 CEL and 1/2 AST. Mechanical stimulation with von Frey hairs was applied to the ipsilateral whisker pad to assess hyperalgesia in naïve (saline; n = 5), complete Freund’s adjuvant (CFA)-inflamed (n = 5), CFA-inflamed rats treated with astaxanthin (AST; 10 mg/kg, i.p.; n = 5), celecoxib (CEL; 10 mg/kg, i.p.; n = 5), or a combination of 1/2 CEL and 1/2 AST (n = 5). #*, p* < 0.05, CFA Day 0 vs. Day1, Day2. *, *p* < 0.05.

Subsequent to CFA administration into the whisker pad, hyperalgesia in rats was evaluated via tactile probing of the injection site and/or facial integument. As presented in Figure 1, CFA induced a substantial decrease in the mechanical escape threshold in inflamed animals, declining from 56.6 ± 3.0 g in naïve controls to 6.8 ± 0.7 g by Day 2 following injection (n = 5, *p* < 0.05; Figure 1). As detailed in preceding investigations, vehicle administration to naïve rats yielded no significant alterations [9,10,11]. Furthermore, the contralateral mechanical threshold within the whisker pad region exhibited no discernible difference between the naïve and inflamed cohorts (58.8 ± 5.1 g vs. 56.7 ± 6.9 g, n = 5, not significant).

### 2.2. The Effect of Administration of AST, CEL and 1/2 CEL + 1/2 AST for Hyperalgesia

Subsequent to daily AST administration, a partial restoration of the mechanical escape threshold was observed in inflamed rats by Day 1 (Figure 1). Figure 1 further demonstrates that the diminished escape threshold in CFA-inflamed rats was recovered to near-baseline levels following AST administration (naïve vs. Day 2 CFA-inflamed + AST: 56.6 ± 3.0 g vs. 49.8 ± 6.1 g, n = 5, not significant). Moreover, the diminished mechanical escape threshold in inflamed rats recovered to baseline levels subsequent to CEL administration on Day 2 (naïve vs. Day 2 inflamed + CEL: 56.6 ± 3.0 g vs. 53.2 ± 6.1 g, n = 5, not significant). Crucially, no discernible difference was observed in the mechanical escape threshold on Day 2 between inflamed cohorts treated with AST and those treated with CEL. Furthermore, the attenuated mechanical escape threshold in inflamed rats was restored to baseline levels subsequent to co-administration of 1/2 CEL + 1/2 AST on Day 2 post-inflammation (naïve vs. Day 2 inflamed + 1/2 CEL + 1/2 AST: 56.6 ± 3.0 g vs. 56.6 ± 3.0 g, n = 5, not significant). Crucially, no statistically significant disparity was observed in the mechanical escape threshold between naïve animals and those receiving the 1/2 CEL + 1/2 AST combination on Day 2.

### 2.3. AST, CEL and 1/2 CEL + 1/2 AST Treatment Reduces Inflammatory Edema: Whisker Pad Thickness Measurements

**Figure 2:**
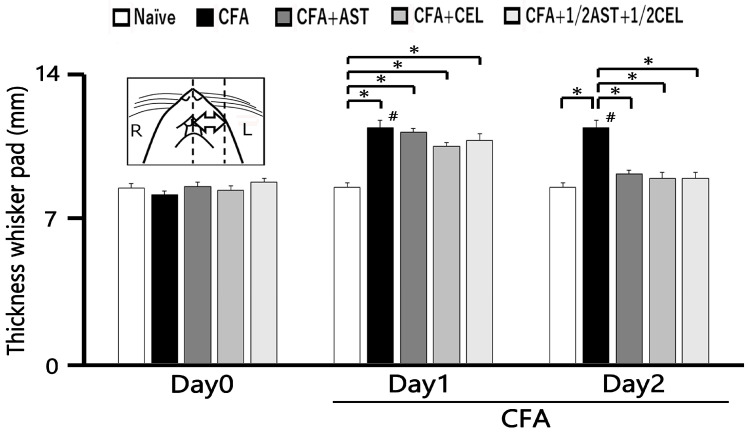
The whisker pad thickness was measured and compared across five experimental groups: naïve, inflamed, inflamed with astaxanthin (AST), inflamed with celecoxib (CEL), and inflamed with 1/2 CEL and 1/2 AST. To quantify the inflammatory response, the thickness of the ipsilateral whisker pad was measured in five groups of rats (n = 5 per group): naïve (saline-treated), complete Freund’s adjuvant (CFA)-inflamed, and CFA-inflamed rats treated with either astaxanthin (AST; 10 mg/kg, i.p.), celecoxib (CEL; 10 mg/kg, i.p.), or a combination of 1/2 CEL and 1/2 AST. Results are expressed as mean ± SEM. *, A significant increase in whisker pad thickness was observed in inflamed groups on Day 1 and Day 2 compared to Day 0 (*p* < 0.05). #, *p* < 0.05, CFA Day 0 vs. Day1, Day2.

Following CFA administration, a statistically significant and persistent elevation in whisker pad edema thickness was observed in inflamed animals relative to naïve controls from Day 1 to Day 2 (*p* < 0.05). On Day 1, the mean thickness of the edematous region measured 11.4 ± 0.4 mm in inflamed rats and 8.6 ± 0.2 mm in naïve controls (n = 5, Figure 2). Crucially, systemic administration of AST, CEL, and their 1/2 + 1/2 combination effectively attenuated the CFA-induced edema, thereby restoring whisker pad thickness to baseline levels by Day 2 (naïve vs. inflamed + AST: 8.6 ± 0.2 mm^2^ vs. 9.2 ± 0.2 mm^2^, n = 5, not significant; Figure 2).

### 2.4. Inflammation-Induced Changes in SpVc WDR Neuron Excitability

**Figure 3:**
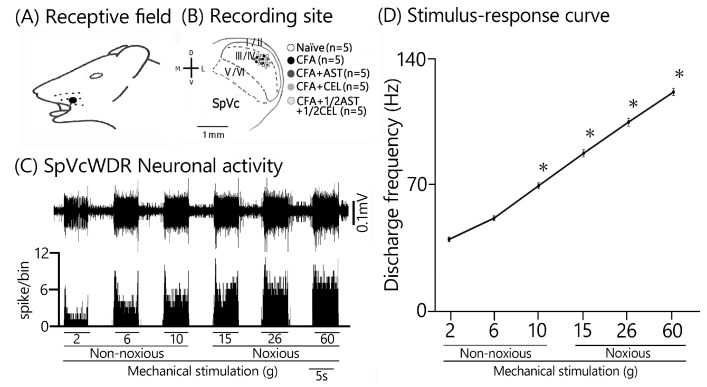
Characterization of SpVc WDR Neuronal Responses to Non-Noxious and Noxious Orofacial Stimuli (**A**) An illustrative receptive field mapping of the whisker pad. (**B**) Somatotopic distribution of SpVc WDR neurons (n = 25) responding to non-noxious and noxious mechanical stimulation of the facial skin. Numbers below each drawing indicate the rostral-caudal position (in mm) relative to the obex. (**C**) Example firing patterns of an SpVc WDR neuron in response to non-noxious (e.g., brush) and noxious (e.g., pinch) mechanical stimulation. (**D**) Stimulus-response curve for SpVc WDR neurons (n = 25), showing firing frequency as a function of stimulus intensity. * *p* < 0.05 for comparison of 2 g vs. 6 g, 10 g, 15 g, 26 g, and 60 g.

Twenty-five SpVc WDR neurons, mechanically activated via whisker pad stimulation, were isolated from rats spanning the naïve, inflamed, inflamed + AST, inflamed + CEL, and 1/2 CEL + 1/2 AST cohorts. Figure 3A illustrates that SpVc WDR neurons, distinguished by their responsiveness to both non-noxious and noxious mechanical stimuli, possessed somatic receptive fields in the whisker pad, primarily situated in layers III-V (n = 23; 92%) and less frequently in layers I-II (n = 2; 8%). Furthermore, their anatomical distribution within the maxillary branch (Figure 3B) demonstrated no salient variations across recording sites among the respective experimental groups. Each SpVc neuron subjected to assessment displayed an augmented firing rate in response to mechanical stimulation of incremental intensities applied to the peak-sensitivity region within its receptive field (Figure 3D). Figure 3D illustrates the stimulus-response function, representing the correlation between mechanical stimulus intensity and the mean discharge frequency of WDR neurons. Consequently, all neurons included in the analysis were characterized as WDR neurons (Figure 3C), consistent with our previously published methodology [9,10,11].

**Figure 4:**
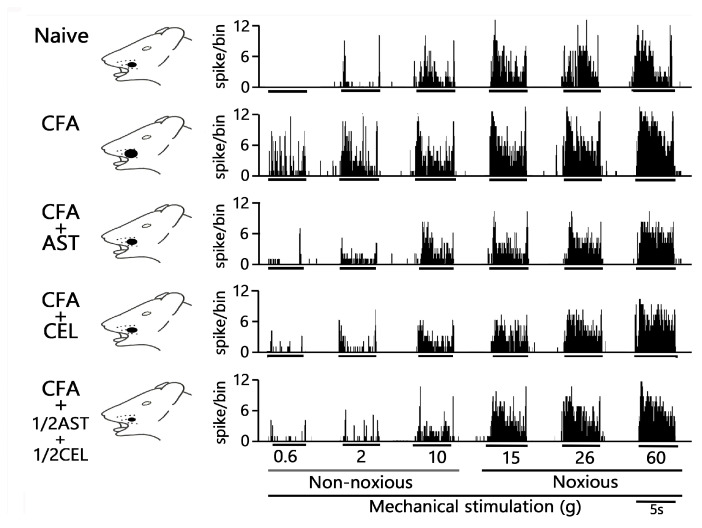
Chronic administration of astaxanthin (AST) or celecoxib (CEL) or 1/2AST and 1/2CEL reverses inflammation-induced hyperexcitability of SpVc WDR neuronal activity in the orofacial region. Representative non-noxious and noxious mechanical stimulation-induced neuronal discharge of SpVc WDR neurons. Data were recorded from naïve (saline; n = 5), complete Freund’s adjuvant (CFA)-inflamed (n = 5), and CFA-inflamed rats treated with either astaxanthin (AST; 10 mg/kg, i.p. for 2 days; n = 5), celecoxib (CEL; 10 mg/kg, i.p. for 2 days; n = 5), or 1/2AST+ 1/2CEL (each at 1/2 dose, i.p. for 2 days; n = 5).

**Figure 5:**
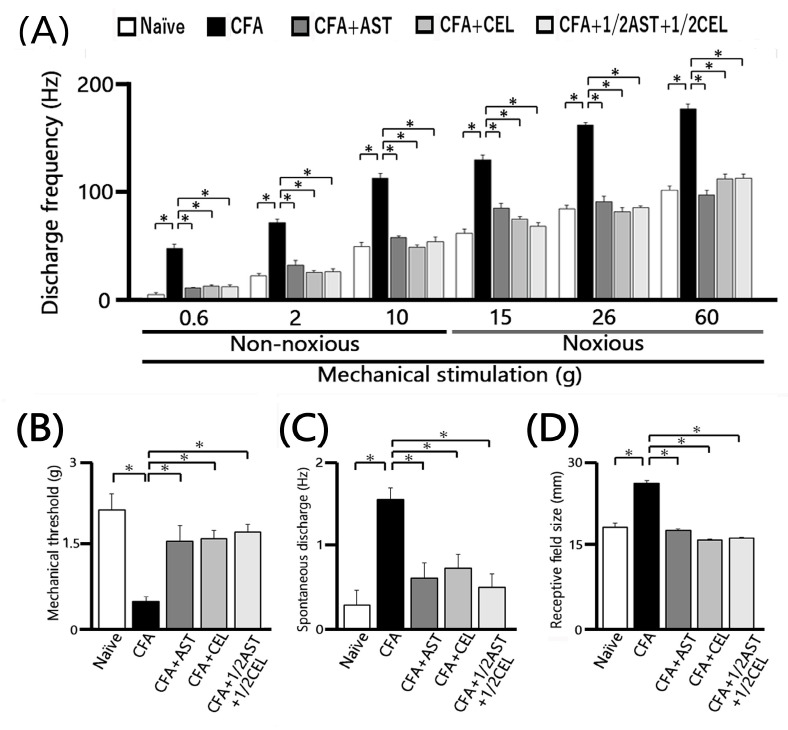
Chronic administration of astaxanthin (AST) or celecoxib (CEL) or 1/2AST and 1/2CEL effectively reverses the inflammation-induced hyperexcitability of SpVc WDR neurons in the orofacial region. (**A**) Comparison of mean discharge frequency of SpVc WDR neurons evoked by non-noxious and noxious mechanical stimulation of the orofacial skin. (**B**) Comparison of mean mechanical threshold of SpVc WDR neurons. (**C**) Spontaneous discharge of SpVc WDR neurons. (**D**) Comparison of mean receptive field size of SpVc WDR neurons. Data in (**A**–**D**) were obtained from naïve (saline; n = 5), complete Freund’s adjuvant (CFA)-inflamed (n = 5), CFA-inflamed rats treated with astaxanthin (AST; n = 5), celecoxib (CEL; n = 5), or 1/2AST and 1/2CEL (n = 5). Data are presented as mean ± SEM. * *p* < 0.05 vs. Naïve group. # *p* < 0.05 vs. Inflamed group.

Consistent with our previous reports [9,10,11], we initially confirmed that CFA treatment resulted in hyperexcitability of SpVc WDR neurons. In naïve rats, spontaneous discharges were recorded in 60% (3/5) of the SpVc neurons examined (Figure 4 and Figure 5C). Spontaneous firing was observed in a subset of naïve neurons, with a mean frequency of 0.3 ± 0.1 Hz (n = 3). Conversely, all WDR neurons in the inflamed group were spontaneously active, firing at a significantly higher mean rate of 1.6 ± 0.1 Hz (Figure 4 and Figure 5C). As previously documented [9,10,11], mechanical stimulation (0.4, 2, 15, 60 g) elicited a greater neuronal response in inflamed rats compared to their naïve counterparts (Figure 4 and Figure 5A). The mean firing rates of SpVc WDR neurons were substantially elevated in the inflamed group relative to the control group (n = 5; Figure 5A). Inflammation significantly decreased the mean mechanical threshold to 0.5 ± 0.1 g in inflamed rats compared with 2.2 ± 0.1 g in naïve controls (n = 5; Figure 5B). We also observed a significantly elevated spontaneous discharge frequency in inflamed rats (Figure 5C). Additionally, the mean receptive field size was significantly larger in the inflamed group (26.6 ± 0.6 mm^2^) than in the naïve group (18.6 ± 0.8 mm^2^) (n = 5, *p* < 0.05; Figure 5D).

### 2.5. Astaxanthin Treatment Prevents the Development of Inflammation-Induced SpVc WDR Neuronal Hyperexcitability

To evaluate the effect of chronic AST administration on SpVc WDR neuronal hyperexcitability, we assessed the behavioral escape threshold in inflamed rats on Day 2. Figure 4 provides representative examples of the discharge frequencies of SpVc WDR neurons in response to both non-noxious (0.6–10 g) and noxious (15–60 g) mechanical stimuli following AST treatment. Subsequent to two days of daily AST administration in inflamed animals, the evoked discharge rates of SpVc WDR neurons, in response to both non-noxious and noxious mechanical stimulation, evinced a reduction to control magnitudes (Figure 4). Specifically, the elevated spontaneous, noxious, and non-noxious firing frequencies, concurrent with the diminished mechanical threshold observed in inflamed rats, reverted to levels comparable to those of control animals. As depicted in Figure 5A, the average discharge frequency of SpVc WDR neurons in inflamed rats was significantly attenuated by AST treatment for both non-noxious and noxious mechanical stimuli (*p* < 0.05). Furthermore, a significant recovery of the mean mechanical stimulation threshold was observed in inflamed rats, reaching control group levels following AST administration (Figure 5B). The spontaneous discharge rate of SpVc WDR neurons in inflamed rats was also significantly suppressed following AST administration (Figure 5C, *p* < 0.05). Furthermore, the mean receptive field dimension in inflamed animals underwent a significant reduction, returning to magnitudes comparable to those of the control cohort (Figure 5D). As delineated in our previous investigations, repeated vehicle administration exerted no appreciable effect on the spontaneous activity or the mechanically (non-noxious/noxious) evoked hyperactivity of SpVc WDR neurons [9,10,11].

### 2.6. Chronic Administration of CEL Inhibits Inflammation-Induced Hyperexcitability of SpVc WDR Neurons in Inflamed Rats

The effect of chronic CEL administration on the hyperexcitability of SpVc WDR neurons was evaluated on Day 2 in inflamed rats. Figure 4 illustrates the reduction in SpVc WDR neuronal firing rates in response to non-noxious and noxious mechanical stimuli following CEL treatment. After two days of continuous CEL administration, the discharge frequency of these neurons in inflamed rats was significantly reduced, returning to levels observed in control animals (Figure 4). CEL administration to inflamed animals resulted in the reversion to baseline levels of the diminished mechanical threshold and augmented spontaneous, noxious, and non-noxious discharge frequencies (*p* < 0.05). The average mechanical stimulation threshold in inflamed rats, following CEL treatment, significantly recovered to levels comparable to those of the control cohort (Figure 5B). Moreover, the spontaneous activity of SpVc WDR neurons in inflamed animals was significantly suppressed after CEL administration (Figure 5C, *p* < 0.05). The mean receptive field dimension in inflamed rats also demonstrated a significant reduction to the magnitudes observed in the control group (Figure 5D).

### 2.7. Chronic Administration of 1/2 AST + 1/2 CEL Inhibits Inflammation–Induced Hyperexcitability of SpVc WDR Neurons in Inflamed Rats

To conclude, we assessed the effect of chronic co-administration of equimolar CEL and AST on the hyperexcitability of SpVc WDR neurons in rats on the second day of inflammation. Figure 4 provides representative examples of SpVc WDR neuronal discharge frequencies elicited by both non-noxious (0.6–10 g) and noxious (15–60 g) mechanical stimuli following treatment with the 1/2 AST + 1/2 CEL regimen in inflamed rats. This treatment restored the diminished mechanical threshold and the augmented spontaneous and evoked discharge frequencies to magnitudes observed in untreated control animals.

Figure 5A demonstrates that the co-administration of 1/2 AST and 1/2 CEL significantly attenuated the mean discharge frequency of SpVc WDR neurons in inflamed rats in response to both non-noxious and noxious mechanical stimuli (*p* < 0.05). This combined therapeutic regimen also led to a significant recovery of the mean mechanical stimulation threshold in inflamed rats to levels comparable to those of the control group (Figure 5B). The combined 1/2 AST + 1/2 CEL regimen significantly attenuated the spontaneous activity of SpVc WDR neurons in inflamed animals (*p* < 0.05; Figure 5C). Lastly, the average receptive field dimension in inflamed rats was significantly reduced, returning to magnitudes observed in the control cohort (Figure 5D). Following the co-administration of equimolar CEL and AST, the median mechanical stimulation threshold in inflamed rats significantly recovered to baseline levels (Figure 5B). Concurrently, the spontaneous discharge frequency of SpVc WDR neurons in inflamed animals was significantly attenuated by the half-dose CEL + half-dose AST regimen (*p* < 0.05; Figure 5C). This combined treatment also resulted in a significant reduction in the mean receptive field size, which attained magnitudes observed in the control cohort (Figure 5D).

## 3. Discussion

### 3.1. AST Exerts a Potent Anti-Hyperalgesic Effect Against Trigeminal Inflammation

We investigated whether systemic AST administration could attenuate inflammation-induced hyperexcitability of SpVc neurons and subsequent mechanical hyperalgesia. The findings of this study include: (i) a significant increase in whisker pad thickness and a reduced threshold for orofacial mechanical escape behaviors in CFA-inflamed rats compared to naïve rats, which aligns with previous reports [9,10,11] (ii) On day 2 following systemic AST administration, CFA-inflamed rats exhibited a reversal of the increased whisker pad thickness and a restoration of the mechanical threshold to control levels; and (iii) vehicle administration had no significant effect on the escape threshold in day 2 CFA-inflamed rats. Previous research has indicated that 10 mg/kg AST inhibits nociceptive behavior in neuropathic pain [24] and reduces PGE2 production under inflammatory conditions [31]. The results from this study corroborate prior research that has indicated that dietary constituents, such as polyphenols and carotenoids, are capable of significantly mitigating CFA-induced mechanical hyperalgesia in a rat model of inflammatory pain [9,10,11]. Collectively, these findings indicate that daily AST administration attenuates inflammation-induced hyperalgesia in the rat whisker pad, likely by suppressing COX-2 signaling and consequently inhibiting PGE2 production through previously described mechanisms [9]

### 3.2. AST Effectively Ameliorates the Pathological Hyperexcitability of SpVc WDR Neurons That Underlies Inflammation-Induced Hyperalgesia

Nociceptive sensory signaling is a widely recognized process consisting of four main steps. It begins with the initial transduction of external stimuli at peripheral terminals, followed by the generation and axonal propagation of action potentials. The signal is then transmitted to central terminals, which serve as presynaptic elements for the initial sensory pathway synapses within the central nervous system [7,32]. While a previous study [31] demonstrated that systemic AST administration dose-dependently inhibits nociceptive behavior (mechanical allodynia and thermal hyperalgesia), a critical gap remains: a systemic in vivo electrophysiological study has not yet been performed, and consequently, the electrophysiological mechanism underlying AST’s suppression of inflammation-induced nociceptive neuronal hyperexcitability is not yet elucidated

Upon peripheral inflammation and/or nerve injury, inflammatory mediators like PGE2 bind to G-protein-coupled E-type prostanoid (EP) receptors. This binding induces the activation of protein kinases A and C (PKA and PKC, respectively) within nociceptive peripheral terminals, which subsequently results in the phosphorylation of mechanosensitive sodium and potassium ion channels and receptors [4,7]. As a result, the activation threshold for transducer channels like transient receptor potential ankyrin 1 (TRPA1) and acid-sensing ion channel 3 (ASIC3) is lowered, and the membrane excitability of the peripheral terminals is enhanced. This drives a higher frequency of action potentials to be conducted to the presynaptic central terminals of the SpVc [4,7]. Consequently, large amounts of glutamate are released into the synaptic cleft, binding to upregulated postsynaptic glutamate receptors. This binding augments excitatory postsynaptic potentials (EPSPs), leading to a barrage of action potentials propagating to higher pain centers and establishing a state of heightened sensitivity, termed peripheral sensitization [4,7].

Our findings demonstrate that daily systemic administration of AST significantly reversed the decreased mechanical stimulation threshold observed in inflamed rats, restoring it to control levels. Furthermore, consistent with these behavioral observations, the mean discharge frequencies of SpVc WDR neurons evoked by both non-noxious and noxious mechanical stimuli in inflamed rats were significantly normalized following chronic AST treatment. Collectively, these results indicate that systemic AST administration effectively mitigates inflammation-induced hypersensitivity of SpVc WDR neuronal activity, presumably through the suppression of peripheral sensitization. This idea was further supported by the observation that systemic administration of AST for two days reversed the increased whisker pad thickness and normalized the reduced mechanical threshold in CFA-inflamed rats to control levels in the present study.

Based on their activation voltage-gated Ca^2+^ channels are divided into two main categories: low-voltage activated (T-type) and high-voltage activated (L, P/Q, N, and R types) [33]. The primary afferent signaling pathway is significantly mediated by both N-type and T-type voltage-gated Ca^2+^ channels. N-type channels, which are activated by high voltage, are predominantly located in the presynaptic regions of laminae I and II of the dorsal horn [33,34,35]. The arrival of an action potential at dorsal root ganglion neurons (including C and Aδ afferents) triggers the opening of these presynaptic N-type calcium channels, thereby initiating the release of nociceptive neurotransmitters such as glutamate, substance P, and calcitonin gene-related peptide (CGRP) onto spinal interneurons and projection neurons [33]. Consistent with prior in vitro findings, astaxanthin was shown to inhibit glutamate release from rat cortex nerve terminals in a dose-dependent fashion, primarily through its ability to suppress presynaptic CaV and MAPK signaling cascades [33]. The inhibitory effect of AST on evoked glutamate release was found to be abolished by N-, P-, and Q-type Ca^2+^ channel blockers [20]. A prior in vitro study [33] found that AST dose-dependently inhibits glutamate release from rat cortical nerve terminals by suppressing presynaptic Ca^2+^ channel and MAPK signaling cascades. Furthermore, the inhibitory effect of AST on evoked glutamate release was abolished by N-, P-, and Q-type Ca^2+^ channel blockers [20]. These results lead us to speculate that systemic AST administration suppresses trigeminal nociceptive neuronal excitability by targeting N-type Ca^2+^ channels at the presynaptic terminals of trigeminal ganglion neurons.

A recent study [21] indicates that AST administration alleviates neuropathic pain by inhibiting NMDA glutamate receptor activity, particularly that of the NMDA receptor subtype 2B (NR2B) protein, which is critically involved in nociception. The NR2B subunit is a key tyrosine-phosphorylated protein, and its phosphorylation has been proposed to increase Ca^2+^ entry through the receptor in both central sensitization and NMDA-dependent synaptic plasticity [36,37]. Thus, it is plausible that AST suppresses glutamatergic excitatory synaptic transmission of SpVc neurons by inhibiting postsynaptic glutamate receptors under pathological pain, including inflammatory conditions.

The present study additionally demonstrated that AST mitigated the inflammation-induced elevation in the mean spontaneous discharge frequency of SpVc WDR neurons. Given that persistent SpVc activity has been associated with spontaneous pain, such as headaches [38], and that this prolonged activation is largely driven by peripheral input [39], our results collectively indicate that AST reduces the heightened spontaneous discharge of whisker-pad-associated SpVc WDR neurons, likely by attenuating peripheral and/or trigeminal ganglion sensitization.

Our previous research indicated that a local GABAergic mechanism modulates nociceptive transmission in SpVc neurons, thereby influencing their mechanical receptive field properties [40]. AST treatment restored the increased receptive field size in inflamed rats to baseline, though the precise mechanisms mediating this effect are yet to be determined. Although the involvement of a GABAergic mechanism in AST’s modulation has not been directly reported, a previous study revealed that natural compounds, including the polyphenolic compound naringenin, exhibit allosteric effects on GABA_A_ receptors, thereby altering their binding affinity [41,42]. Therefore, AST may regulate local GABAergic tonic control over nociceptive mechanoreceptive signaling and suppress central excitatory synaptic transmission within that pathway. Our previous research showed that local iontophoretic administration of GABA_A_ receptor agonists and antagonists altered the mechanical receptive field size [40]. It is plausible that AST application may reduce receptive field sizes by activating GABAergic inhibitory mechanisms in the SpVc local circuit, though this hypothesis requires further investigation.

To the best of our knowledge, while sex-related differences in AST’s effect on the aging brain have been reported [43], no studies have investigated its impact on pain sensitivity across sexes. A prior investigation observed that experimental pain perception varies during the menstrual cycle, with increased sensitivity to several pain modalities during the luteal phase relative to the follicular phase [44]. While sex hormones are commonly acknowledged to play a major role in pain variability, the detailed reasons for this disparity remain unclear [45]. Therefore, we only examined our hypothesis in male rats, and further studies are needed to elucidate any sex-related differences.

### 3.3. Contribution of AST to Hyperalgesia Alleviation Through SpVc Neuronal Hyperexcitability Suppression

CAM therapies, such as herbal medicines and acupuncture, are frequently employed for pain management when conventional treatments prove ineffective [8]. Research has shown that chronic administration of dietary constituents, including polyphenols and carotenoids, can mitigate inflammation-induced mechanical hyperalgesia by suppressing SpVc WDR neuronal hyperexcitability through both peripheral and central COX-2 cascade signaling pathways [9,10,11]. AST demonstrates superior antioxidant activity relative to other carotenoids like lutein and zeaxanthin [13,14,15] and possesses a wide range of biological activities, including anti-inflammatory, antitumor, anti-diabetic, and immunomodulatory properties [16,17,18,19]. Previous research has demonstrated that potent antioxidants, including AST, possess anti-inflammatory properties across various organs and tissues. These effects are primarily mediated through the nuclear factor erythroid 2-related factor 2 (Nrf2) and nuclear factor kappa B (NF-κB) signaling pathways [31,46].

This study identified several key effects of CEL and 1/2 CEL + 1/2 AST administration: (i) Restoration of the mechanical threshold in inflamed rats to control levels by day 2 of chronic treatment. (ii) Significant reduction in the mean discharge frequency of SpVc WDR neurons to both non-noxious and noxious mechanical stimuli in inflamed rats. (iii) Significant decrease in the elevated mean spontaneous discharge of SpVc WDR neurons observed in inflamed rats. (iv) Normalization of the expanded mean receptive field size in inflamed rats. Our findings indicate that AST-mediated inhibition of hyperalgesia-associated SpVc neuronal hyperexcitability was comparable in magnitude to that of CEL (10 mg/kg, i.p.), a specific COX-2 blocker. These findings indicate that AST may serve as a promising therapeutic agent for alleviating trigeminal inflammatory mechanical hyperalgesia, particularly in conditions like temporomandibular joint disorders and postoperative pain [4,7]. This suggests a potential role for AST in CAM-based treatments. The notion was also reinforced by the finding that AST alone produced an inhibition of hyperalgesia-associated SpVc neuronal hyperexcitability that was similar in magnitude to the effects observed with 1/2 CEL + 1/2 AST administration.

**Figure 6:**
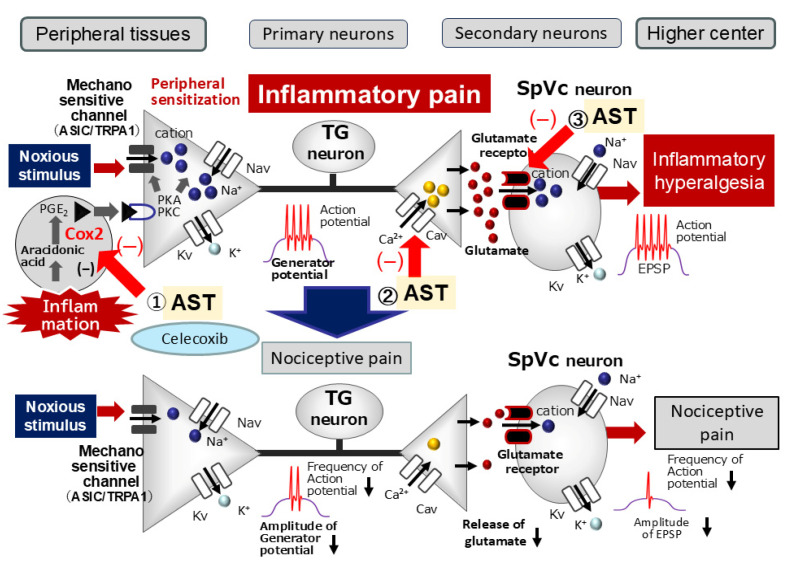
Proposed Mechanisms by Which Astaxanthin (AST) Suppresses Inflammation-Induced Mechanical Hyperalgesia. When systemically administered, AST is hypothesized to attenuate inflammation-induced mechanical hyperalgesia primarily by suppressing the hyperexcitability of SpVc wide-dynamic range (WDR) neurons. This effect is possibly mediated via multiple pathways: (i) Peripheral Action: Inhibition of the peripheral cyclooxygenase (COX)-2 cascade signaling pathways, which would decrease the firing frequency of action potentials in the nociceptive nerve terminals; (ii) Central Action: Decreased activation or expression of postsynaptic glutamate receptors and reduced activity of Cav channels at the central terminals of nociceptive neurons in the SpVc. These combined actions are proposed to inhibit the conduction of pain signals to the SpVc and higher pain centers, thereby contributing to the attenuation of hyperalgesia and modulating both the discriminative and affective-motivational aspects of pain.

While NSAIDs are commonly prescribed for symptom management, they are known to have detrimental effects in orthodontic patients, including reduced tooth movement. This is attributed to their inhibition of PGE_2_, a key mediator of osteoclast-mediated bone remodeling [47,48]. The mechanical forces applied during orthodontic treatment to move teeth frequently lead to gingival inflammation and bone resorption on the tension side [49]. Recent investigations have shown that prolonged administration of the phytochemical resveratrol effectively mitigates mechanical, ectopic hyperalgesia induced by tooth movement and is correlated with decreased SpVc WDR neuronal hyperexcitability in anesthetized rats [50]. Consequently, these findings suggest that this dietary component holds therapeutic potential for ectopic pain, exemplified by the discomfort associated with orthodontic treatment [50]. The present study further demonstrated that systemic administration of a half-dose of AST effectively substituted for a half-dose of CEL. These results indicate that AST administration is a promising strategy for attenuating orthodontic treatment-induced ectopic hyperalgesia, potentially without the adverse effects associated with NSAIDs. However, comprehensive investigations are warranted to fully establish this potential. This study is the first to directly compare the suppressive potency of AST and the specific COX-2 antagonist CEL on inflammation-induced SpVc neuronal excitability, which is a key contributor to hyperalgesia. As summarized in Figure 6, we propose that systemic AST attenuates inflammation-induced mechanical hyperalgesia primarily by suppressing SpVc WDR neuronal hyperexcitability, an effect mediated by the inhibition of peripheral COX-2 cascade signaling. Additionally, AST’s effects on Cav channels and glutamate receptors may decrease the firing frequency of action potentials in nociceptive nerve terminals, thereby inhibiting pain signal conduction to the SpVc and higher centers. These findings therefore provide a foundation for the development of novel analgesic drugs for the treatment and prevention of trigeminal inflammatory pain, including clinical orofacial pain, with a potentially lower incidence of side effects.

## 4. Materials and Methods

All experimental procedures detailed herein received authorization from the Animal Use and Care Committee of Azabu University (No. 230120-11) and conformed to the ethical principles stipulated by the International Association for the Study of Pain [51]. Stringent measures were implemented to minimize both the number of animals utilized and their potential distress. Additionally, all experimental manipulations were performed by investigators blinded to the treatment conditions.

### 4.1. Experimental Procedures: Inflammation Induction and Drug Administration (AST and NSAIDs)

Male Wistar rats, ranging from 210 to 260 g in body weight, were maintained on a 12 h light/dark cycle (lights on: 07:00–19:00). While variations in experimental pain responses between sexes have been observed, the specific mechanisms underpinning these disparities remain incompletely characterized [45]. Building on previous observations regarding sex differences in pain responses, the present study was conducted using exclusively male Wistar rats. Twenty-five adult male rats were allocated into five groups (n = 5 per group): (i) Naïve (ii) Inflamed (iii) Inflamed + AST (10 mg/kg, i.p.; Sigma-Aldrich, Milano, Italy) (iv) Inflamed + CEL (10 mg/kg, i.p.; Sigma-Aldrich, Milano, Italy) (v) Inflamed + CEL (5 mg/kg, i.p.; Sigma-Aldrich, Milano, Italy) + AST (5 mg/kg, i.p.). Consistent with prior findings, these concentrations of AST and CEL significantly suppressed COX-2 activity in vitro [24,30]. For the induction of inflammation, each animal was initially anesthetized with 3% isoflurane, followed by the administration of 0.05 mL of CFA (a 1:1 oil/saline mixture) into the left facial skin, consistent with our established protocol [9,10,11]. Control naïve animals received an equivalent volume of vehicle (0.9% NaCl) at the corresponding facial site. Both AST and CEL were solubilized in dimethyl sulfoxide (DMSO).

Naive control animals received an injection of 0.9% NaCl saline solution into the left facial skin. AST and CEL were solubilized in DMSO and administered systemically to the rats over a two-day period. Prior to each daily compound administration, behavioral assessments were performed. To quantify the efficacy of AST in mitigating peripheral inflammation, the thickness of CFA-induced edema in the whisker pad was measured in all experimental cohorts, as described elsewhere [9]. In parallel with the behavioral analysis, electrophysiological experiments were conducted on Day 2 and were limited to the following groups: naïve, CFA-inflamed, CFA-inflamed + AST, CFA-inflamed + CEL, and AST 1/2 + CEL 1/2.

### 4.2. Assessment of Mechanical Withdrawal Threshold

The mechanical threshold of escape behavior was determined as previously described [9,10,11]. To assess mechanical hyperalgesia, we applied an ascending series of von Frey hairs (Semmes-Weinstein Monofilaments, North Coast Medical, CA, USA) to both the ipsilateral and contralateral facial skin regions one to two days following CFA or vehicle administration. Each stimulus was applied three times with a 5 s inter-stimulus interval. The escape threshold was defined as the lowest force that elicited a head-withdrawal response in at least one of the three applications.

### 4.3. Electrophysiological Analysis of SpVc WDR Neurons: Single-Unit Recording

Two days after CFA or vehicle injection, electrophysiological recordings were performed as previously described. Electrophysiological data were collected from 25 adult male Wistar rats. Each rat was initially sedated with 3% isoflurane, then anesthetized with a mixture of medetomidine (0.3 mg/kg), midazolam (4.0 mg/kg), and butorphanol (5.0 mg/kg). Supplementary doses of the anesthetic mixture (0.25–0.45 mL/kg/h) were administered as needed through a cannula in the jugular vein to maintain anesthesia. Electrophysiological recordings commenced two days post-administration of CFA or vehicle, following established procedures. A total of 25 adult male Wistar rats underwent these electrophysiological assessments. Anesthesia was initially induced with 3% isoflurane. Subsequent anesthetic maintenance was achieved via continuous intravenous infusion (through a jugular vein cannula) of a tailored mixture (0.3 mg/kg medetomidine, 4.0 mg/kg midazolam, and 5.0 mg/kg butorphanol), with supplemental doses administered at 0.25–0.45 mL/kg/h as deemed necessary. To ensure adequate anesthesia, the absence of a withdrawal response to a paw pinch was confirmed. Rectal temperature was precisely regulated at 37.0 ± 0.5 °C during the entire recording session via a homeothermic blanket (Temperature Controller 40-90-8D; FHC Aspen, Tokyo, Japan). Consistent local analgesia was ensured at all surgical wound margins via the application of 2% lidocaine solution (Xylocaine). Subsequent to positioning within a stereotaxic apparatus (SR-50; Narishige, Tokyo, Japan), a midline incision and separation of the neck musculature provided access to the medullary brainstem, which was further exposed by careful incision of the atlanto-occipital ligament and dura mater. Extracellular recordings of single-unit activity within the ipsilateral SpVc were conducted utilizing a tungsten microelectrode (3–5 MΩ). Electrode positioning was precisely controlled via a micromanipulator (SM-11 and MO-10; Narishige, Tokyo, Japan), with adjustments made in 10 µm increments or decrements, guided by the stereotaxic coordinates established in the Paxinos and Watson rat brain atlas [52]. Neural signals subsequently underwent amplification (DAM80; World Precision Instruments, Sarasota, FL, USA), band-pass filtering (0.3–10 KHz), real-time monitoring with an oscilloscope (SS-7672; Iwatsu, Tokyo, Japan), and digital acquisition for post hoc analysis using Power Lab hardware and Chart v.5 software (AD Instruments, Oxford, UK), consistent with prior methodology [9,10,11].

### 4.4. Experimental Design and Protocols

The characterization of extracellular single-unit SpVc WDR neuronal responses to mechanical stimulation of the whisker pad was conducted as detailed below. To preclude the sensitization of peripheral mechanoreceptors, a paintbrush served as a rapid probing instrument to roughly delineate the receptive field on the ipsilateral whisker pad. Following this, the left whisker pad was explored for single units exhibiting responses to a spectrum of von Frey monofilaments, encompassing both non-noxious (0.2, 0.6, 2, 6, 10 g) and noxious (15, 26, 60 g) mechanical stimuli. Each stimulus was presented for 5 s, with an inter-stimulus interval of 5 s [9,10,11]. The criteria for identifying WDR neurons included a graded response to both non-noxious and noxious mechanical stimuli applied within a defined receptive area. Following the identification of a nociceptive SpVc WDR neuron responsive to the whisker pad, we ascertained its mechanical stimulation threshold and precisely documented the dimensions of its receptive field. The mechanical receptive fields of these neurons were mapped by systematically applying von Frey hairs to the facial skin and transcribing the responsive contours onto a life-sized schematic of the rat using tracing paper [9,10,11]. Quantification of WDR neuronal activity in response to mechanical stimulation involved subtracting baseline neuronal activity from stimulus-evoked activity. Spontaneous discharge frequencies were evaluated over 2–5 min intervals. The rationale for focusing on WDR neurons in this investigation is supported by prior findings: (i) SpVc WDR neurons are critically implicated in the mechanisms of mechanical hyperalgesia [4,7]; and (ii) evidence suggests a potential conversion of nociceptive specific (NS) neurons to WDR neurons following CFA-induced inflammation [3,4]. Therefore, our study focused exclusively on the effects of AST on nociceptive SpVc WDR neuronal activity, while intentionally omitting the investigation of NS neurons. Peristimulus histograms, with a bin size of 100 ms, were constructed for each stimulus presentation. The mean spontaneous and mechanically evoked discharge frequencies, in addition to the mean mechanical thresholds, of SpVc WDR neurons were assessed to comparative analysis across the following five experimental cohorts: naïve, CFA-inflamed, CFA-inflamed + AST, CFA-inflamed + CEL, and CFA-inflamed + 1/2 AST + 1/2 CEL. The precise anatomical locations of single-unit recordings within the SpVc were ascertained from micromanipulator readouts, detailing coordinates relative to the obex, midline, and dorsal medullary surface. These coordinates were cross-referenced with the rat brain atlas, aligning with methodologies established in our prior investigations [9,10,11,52].

### 4.5. Statistical Analysis

All reported values represent the mean ± SEM. A one-way repeated measures analysis of variance was utilized for statistical analysis and subsequent Tukey–Kramer’s post hoc tests and Student’s *t*-test were performed on the behavioral and electrophysiological data (Excel Statcel 4). A threshold of *p* < 0.05 was established for statistical significance.

## 5. Conclusions

In the present study, we provide evidence that systemic administration of AST attenuates inflammatory mechanical hyperalgesia associated with hyperexcitability of nociceptive SpVc WDR neurons via inhibition of the COX-2 signaling pathway. These findings support the proposed potential of AST as a therapeutic agent for CAM strategies, potentially offering an alternative to NSAID for preventing trigeminal inflammatory mechanical hyperalgesia

## Data Availability

All data from this study are included in the main body of the article.

## Abbreviations

The following abbreviations are used in this manuscript:

AST	Astaxanthin
NSAID	Non-steroidal anti-inflammatory drug
CEL	Celecoxib
SpVc	Spinal Trigeminal nucleus caudalis
WDR	Wide-dynamic range
TG	Trigeminal ganglion
CFA	Complete Freund’s Adjuvant
CAM	Complementary Alternative Medicine
COX-2	Cyclooxygenase-2
PGE_2_	Prostaglandine E2
MAPK	Mitogen activated protein kinase
PKA	Protein Kinase A
PKC	Protein Kinase C
NMDA	N-methyl-D-aspartate
NR2B	NMDA receptor subtype 2B
Nav	Voltage-gated Na channels
Kv	Voltage-gated K channels
Cav	Voltage-gated Ca channels
TRPA1	Transient receptor protein ankyrin 1
ASIC3	Acid sensing ion channels 3
EPSP	Excitatory Post synaptic Potential
EP	Prostanoid E
